# Fgf10 and Sox9 are essential for the establishment of distal progenitor cells during mouse salivary gland development

**DOI:** 10.1242/dev.146019

**Published:** 2017-06-15

**Authors:** Lemonia Chatzeli, Marcia Gaete, Abigail S. Tucker

**Affiliations:** 1 Centre for Craniofacial and Regenerative Biology, Department of Craniofacial Development & Stem Cell Biology, King's College London, London SE1 9RT, UK; 2 Department of Anatomy, Faculty of Medicine, Pontificia Universidad Católica de Chile, Santiago 8331150, Chile

**Keywords:** Branching morphogenesis, Epithelial progenitors, Sox9, Fgf signalling, Salivary glands

## Abstract

Salivary glands are formed by branching morphogenesis with epithelial progenitors forming a network of ducts and acini (secretory cells). During this process, epithelial progenitors specialise into distal (tips of the gland) and proximal (the stalk region) identities that produce the acini and higher order ducts, respectively. Little is known about the factors that regulate progenitor expansion and specialisation in the different parts of the gland. Here, we show that *Sox9* is involved in establishing the identity of the distal compartment before the initiation of branching morphogenesis. *Sox9* is expressed throughout the gland at the initiation stage before becoming restricted to the distal epithelium from the bud stage and throughout branching morphogenesis. Deletion of *Sox9* in the epithelium results in loss of the distal epithelial progenitors, a reduction in proliferation and a subsequent failure in branching. We demonstrate that *Sox9* is positively regulated by mesenchymal *Fgf10*, a process that requires active Erk signalling. These results provide new insights into the factors required for the expansion of salivary gland epithelial progenitors, which can be useful for organ regeneration therapy.

## Abstract (precis)

**Summary:** Deletion of Sox9 in the salivary gland epithelium results in loss of the distal epithelial progenitors, a reduction in proliferation and a subsequent failure in branching in mice.

## INTRODUCTION

To develop therapeutic strategies for organ regeneration, we first need to understand how progenitor cells contribute to organ formation. During development, organs such as lungs, lacrimal glands, pancreas and salivary glands undergo branching morphogenesis, a process that efficiently increases the surface area with a minimum increase of volume. Common to all branching epithelium, the embryonic salivary gland epithelium starts as a placode (also known as the prebud), which then elongates leading to the formation of a stalk attached to the bud (also known as the initial bud). The epithelium then undergoes sequential rounds of epithelial budding, clefting and epithelial outgrowth creating a highly branched network divided into ducts and endbuds (Affolter et al., 2003), these endbuds forming the secretory acini of the adult gland. Branching morphogenesis in many organs has been shown to require constant interactions between the epithelium, mesenchyme, blood vessels and nerves (Knosp et al., 2012). Salivary glands have long been used as a model to study branching morphogenesis because of their ease of *ex vivo* manipulation (Tucker, 2007). Among the three major types – submandibular (SMG) secreting seromucous saliva, sublingual (SL) secreting mucous saliva, and parotid (PG) secreting serous saliva – the SMG is the most commonly studied.

As the epithelium initiates and undergoes branching it becomes specialised into distinct epithelial compartments. In salivary glands, the earliest stage reported for this specialisation is after the initiation of branching at the pseudoglandular stage [embryonic day (E) 13.5] (Lombaert et al., 2011, 2013; Knox et al., 2010; Arnold et al., 2011). Based on the position of cells within the developing gland and the expression of progenitor markers, the epithelium is divided into proximal and distal progenitors. In salivary glands, the proximal progenitors, the cells located closer to the oral epithelium at the stalk region, express markers such as cytokeratin 5 (K5; also known as keratin 5, Krt5) and *Sox2* [SRY (sex-determining region Y)-box 2] (Lombaert et al., 2011; Knox et al., 2010; Arnold et al., 2011). The distal progenitors, located at the end of the gland, express cytokeratin 14 (K14; also known as keratin 14, Krt14), *Kit* and *Sox10* (Lombaert et al., 2013). *Myb* is also expressed by the distal epithelial progenitors, as shown at E17.5 when terminal differentiation starts to occur (Matsumoto et al., 2016). The location of epithelial progenitors at specific time points during development has been suggested to determine their progeny. When epithelial rudiments of E13.5 SMGs were cultured *ex vivo* with Fgf7 and Fgf1, the distal epithelial progenitors labelled after a day in culture contributed to the formation of acini (secretory cells producing saliva) and secondary- and tertiary-branched ducts. However, when labelled after 3 days in culture at a stage when pro-acinar differentiation had already initiated, their lineage was restricted to the acinar compartments. The more proximal progenitors, on the other hand, could only contribute to the formation of higher order branched ducts (Matsumoto et al., 2016). Lumen formation in the ducts is marked by F-actin deposition whereas acinar differentiation is marked by the expression of Mist1 (bHLHa15) (Walker et al., 2008; Aure et al., 2015). Interestingly, distal epithelial progenitors have been shown to be more proliferative than proximal progenitors (Steinberg et al., 2005; Matsumoto et al., 2016).

Although there is increasing information on the factors that regulate salivary gland branching morphogenesis, little is known about the signals that control the expansion of the different epithelial progenitors, or whether the distal epithelial progenitors alone are required for branching morphogenesis. Acetylcholine signalling through the parasympathetic ganglion was shown to promote the expansion of K5^+^ cells and their differentiation to the ductal K19 (Krt19)^+^ lineage by a process that required epidermal growth factor receptor (EGFR) signalling (Knox et al., 2010). On the other hand, epithelial Wnt and Fgf receptors in combination with Kit signalling were shown to promote the expansion of the distal *Sox10^+^K14^+^* population (Lombaert et al., 2013; Matsumoto et al., 2016). Key pathway components for Fgf signalling in developing salivary glands are *Fgf10* and its receptor *Fgfr2*, as mutations in either of these two genes lead to an arrest of salivary gland development at the placode stage (Jaskoll et al., 2005). *Fgf10* is expressed in the neural crest-derived mesenchyme that surrounds the gland, with conditional knockout of *Fgf10* in the neural crest mimicking the null phenotype (Teshima et al., 2016a), whereas *Fgfr2* is expressed in the gland epithelium (Jaskoll et al., 2002). Similar to salivary glands, other branching organs were also arrested after knockout of *Fgf10*, including the lung and lacrimal glands, and the pancreas was hypoplastic (Ohuchi et al., 2000).

In the lungs, lacrimal glands and pancreas, Fgfr2 signalling has been shown to regulate the expression of *Sox9*, which appears to act as a distal epithelial marker (Abler et al., 2009; Chang et al., 2013; Chen et al., 2014; Seymour et al., 2012). Sox9 is a transcription factor that belongs to the highly conserved SOX family (subgroup E) characterised by the presence of the high mobility group DNA-binding domain of SRY (Pritchett et al., 2011). Initially *Sox9* was identified as a gene linked to campomelic dysplasia, a syndrome that causes male-to-female sex reversal and skeletal defects (Wagner et al., 1994). Apart from its importance in gonadal formation and chondrogenesis, *Sox9* is expressed in the epithelium of many developing branching organs, including lacrimal glands, lungs, pancreas and kidneys. Its requirement for their development varies as conditional *Sox9* inactivation results either in complete agenesis, as in the case of the lacrimal glands (Chen et al., 2014), or in hypoplasticity, as in the case of the lungs and pancreas (Chang et al., 2013; Rockich et al., 2013; Seymour et al., 2007). Kidneys also rely on *Sox9* expression for their development; however, the severity of the phenotype is variable and ranges from agenesis to hypoplasia (Reginensi et al., 2011). Despite these variabilities, in general epithelial *Sox9* expression has been shown to promote progenitor cell expansion and extracellular matrix (ECM) deposition (Chang et al., 2013; Rockich et al., 2013; Chen et al., 2014). qPCR has shown that *Sox9* is expressed in developing salivary glands, with a peak of expression at E15.5 (Lombaert and Hoffman, 2010). The role of *Sox9* in salivary glands, however, has not been assessed.

Here, we investigated the importance of Sox9 in salivary gland development using the *Sox9^flox/flox^; K14-Cre^+^* (*Sox9^CKO^*) mouse line, in which *Sox9* is ablated in epithelial tissues from the initiation stage of salivary gland development. We find that *Sox9* is highly expressed in the distal epithelial progenitors where it is required for their specification as a distal epithelial population and for subsequent branching morphogenesis. *Sox9* expression is maintained by *Fgf10* signalling by a process that requires active Erk signalling.

## RESULTS

### *Sox9* is restricted to the distal epithelial compartment from the bud stage of development and is maintained in this region throughout development

**Fig. 1.**
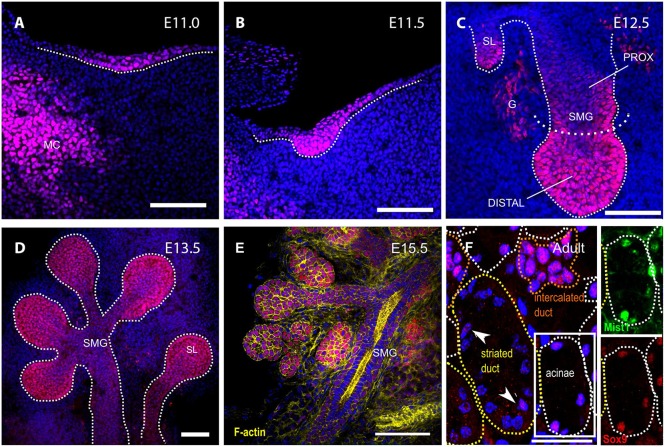
**Sox9 is expressed throughout the development of** **the** **submandibular gland.** (A-F) Sox9 immunofluorescence (red) at the placode [E11.0 (A), E11.5 (B)], initial bud [E12.5 (C)], pseudoglandular [E13.5 (D)], canalicular [E15.5 (E)] and adult (F) stages. DNA is shown in blue (DAPI), F-actin in yellow and Mist1 in green. Dotted lines in A-D delineate the salivary gland epithelium. Insets in F show magnifications of an acinus stained for Mist1 (green) and Sox9 (red). Arrowheads point to Sox9-positive cells within the striated duct. G, ganglion; MC, Meckel's cartilage; SL, sublingual gland; SMG, submandibular gland. Scale bars: 100 μm.

**Fig. 2.**
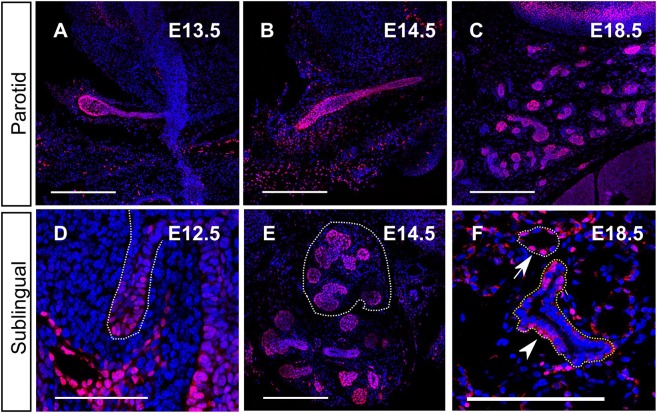
**Sox9 expression in the parotid and sublingual gland is similar to the submandibular.** (A-F) Sox9 immunofluorescence (red) in the parotid (A-C) and sublingual (D-F) glands at the bud stage (A,D) and at E14.5 (B,E) and E18.5 (C,F). Dotted white lines in D and E outline the sublingual glands. The white dotted line in F outlines an acinus and the yellow a duct. Arrow points to Sox9^+^ cells in the acinus and the arrowhead points to Sox9^+^ cells in the duct. DNA is shown in blue (DAPI). Scale bars: 250 μm (A-C,E,F); 50 μm (D).

As a first approach to understanding the function of *Sox9*, we traced its protein distribution during SMG development. During all stages, Sox9, as expected for a transcription factor, was detected in the nucleus and was absent from the epithelium of *Sox9^CKO^* glands, indicating the high specificity of this antibody for Sox9 (Fig. 1; Fig. S1B). At gland initiation (E11.0-11.5), all the epithelial cells of the placode and the early invaginating bud were Sox9^+^ (Fig. 1A,B). However, at the bud stage (E12.5), high levels of Sox9 expression were only observed distally at the tip of the buds, with much lower expression proximally next to the oral surface (Fig. 1C). This pattern of epithelial Sox9 expression, with higher levels at the distal tips, was maintained throughout branching morphogenesis at E13.5 and E15.5 (Fig. 1D,E). As lumens started to form in the more distal ducts (as indicated by F-actin staining), Sox9 expression turned off (Fig. 1E). Interestingly, in the adult when differentiation had fully occurred, Sox9^+^ cells were still predominantly located in more distal structures, with large numbers of positive acinar cells, as shown by co-expression of Sox9 and Mist1 (Fig. 1F, insets). All Mist1^+^ cells were also Sox9^+^, indicating an important link between these two transcription factors in adult glands. Expression of Sox9 was also observed in the small distally located intercalated ducts (Fig. 1F, yellow outline), whereas fewer cells that stained less intensely were found in the bigger more proximal ducts (Fig. 1F, arrowheads). Interestingly, the intensity of Sox9 appeared to be lower in the acinar compartments than in the intercalated ducts in the adult (Fig. 1F), suggesting a change in role in fully differentiated glands. The PG (Fig. 2A-C) and SL (Fig. 2D-F) glands displayed a similar pattern of Sox9 expression compared with the SMG, suggesting that Sox9 plays a similar developmental role in all of the three major salivary glands (Fig. 2).

### Sox9^+^ epithelial cells are progenitors of the entire salivary gland epithelium

**Fig. 3.**
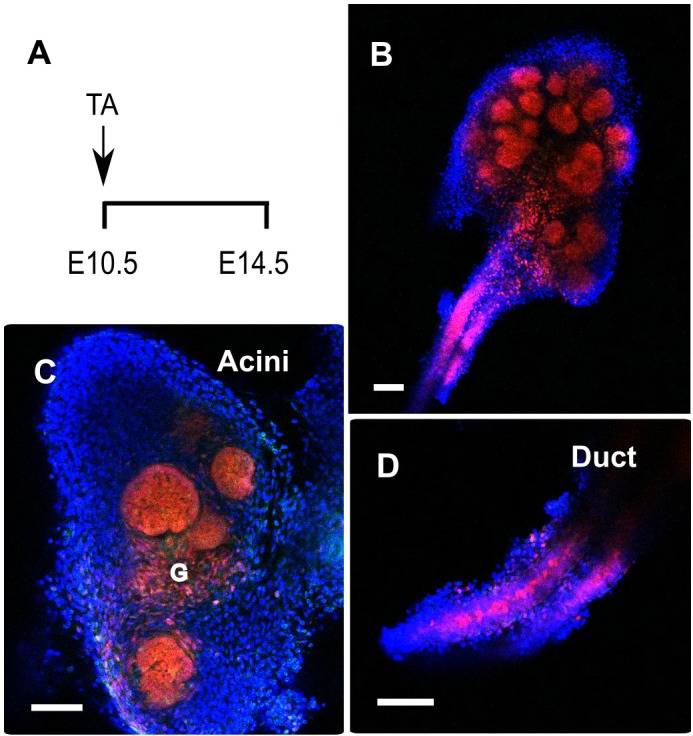
**Sox9-positive cells are progenitors of the entire submandibular gland epithelium.** (A) Experimental strategy used to follow the progeny of Sox9-expressing cells with the *Sox9-creERT2; R26-tdTomato* line. Tamoxifen (TA) was given at E10.5 and embryos were collected at E14.5. (B-D) Tomato-labelled cells (red) were detected in the whole submandibular gland at E14.5, (B) in the acini (C) and in the duct (D). G, ganglion. Scale bars: 100 μm.

Our protein localisation analysis revealed early Sox9 expression in the placode epithelium. To investigate whether these early Sox9^+^ epithelial cells act as progenitors, we traced their progeny using *Sox9-creERT2* mice crossed with *Rosa-tdTomato* mice. After tamoxifen administration at E10.5, the entire epithelium of the E14.5 submandibular gland was labelled in red including all the ductal and acinar structures (Fig. 3). In agreement with the early Sox9 expression in the ganglia (Fig. 1C), label was also detected in the ganglia cells found in close association with the submandibular gland epithelium (Fig. 3C). Earlier tamoxifen administration labelled the mesenchyme (date not shown) in agreement with Sox9 expression in the neural crest cells that form the salivary gland mesenchyme (Zhao et al., 1997).

### *Sox9* is required for the formation of distal epithelial progenitors and for branching morphogenesis

To assess the role of *Sox9* during salivary gland development, we deleted *Sox9* flox alleles in the oral epithelium using *K14-Cre*. The *K14* promoter induces Cre recombination in almost all epithelial cells of the salivary gland from the initiation stage (Fig. S1A,B). Immunofluorescence for Sox9 confirmed almost complete loss of Sox9 in the salivary gland epithelium, although a very small number of cells remained positive for Sox9, both at the placode (Fig. S1A,B) and later at the bud stage (Fig. S1C,D). As expected, Sox9 was still expressed in the surrounding mesenchyme, including Meckel's cartilage and the ganglia (Fig. S1B,D).

**Fig. 4.**
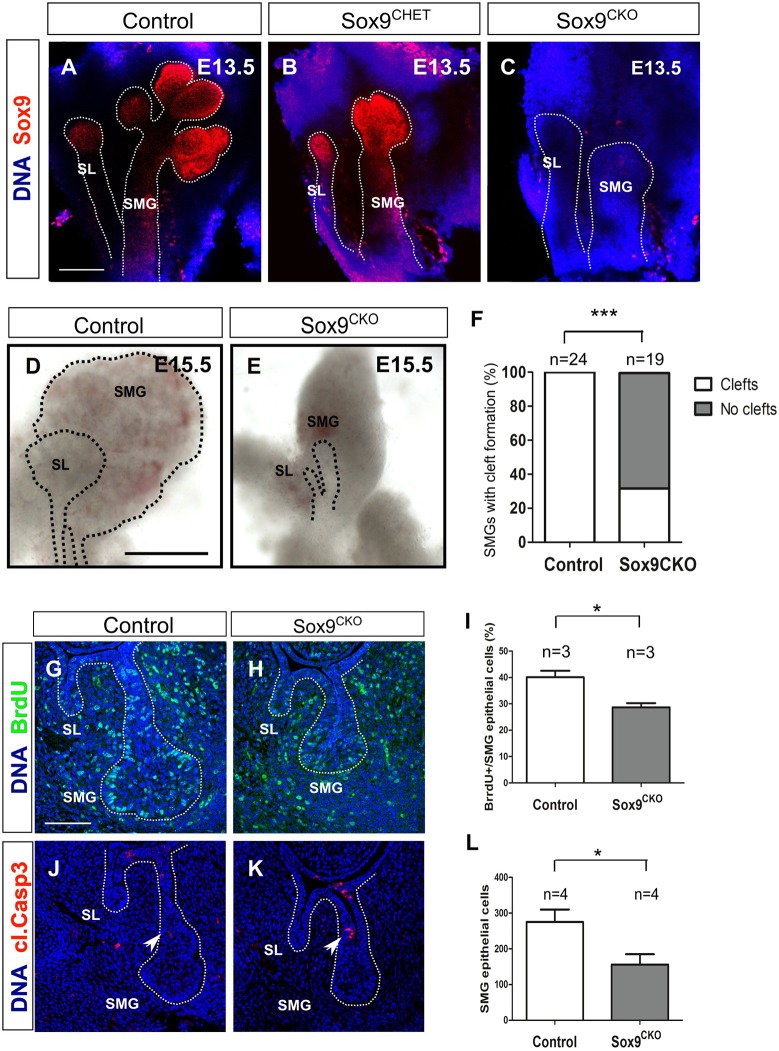
**Sox9 is required for branching morphogenesis.** (A-C) Sox9 immunofluorescence (red) in control (A), *Sox9^CHET^* (B) and *Sox9^CKO^* (C) at the pseudoglandular stage (E13.5). (D,E) Submandibular and sublingual glands dissected from control (D) and *Sox9^CKO^* (E) mice at E15.5. (F) Quantification of cleft formation in the control and *Sox9^CKO^* submandibular glands at the pseudoglandular stage (E13.5). ‘n’ equals the number of submandibular glands. ***P<0.0001. (G,H) BrdU immunofluorescence (green) in control (G) and *Sox9^CKO^* (H) submandibular glands at the bud stage (E12.5). (I) Quantification of the percentage of epithelial BrdU^+^ cells in the control and *Sox9^CKO^* submandibular glands at the bud stage (E12.5). (J,K) Cleaved caspase 3 immunofluorescence (red) in control and *Sox9^CKO^* submandibular glands at the bud stage (E12.5). Arrowheads indicate apoptotic cells at the stalk region. (L) Quantification of epithelial cell number in control and *Sox9^CKO^* submandibular glands at the bud stage (E12.5). Dotted lines in A-E,G,H,J,K delineate the salivary gland epithelium. Error bars in I and L represent s.e.m.; *P<0.05. DNA is shown in blue (DAPI) for A-C and G,H,J,K. SL, sublingual gland; SMG, submandibular gland. Scale bars: 200 μm (A-C); 500 μm (D,E); 100 μm (G,H,J,K).

**Fig. 5.**
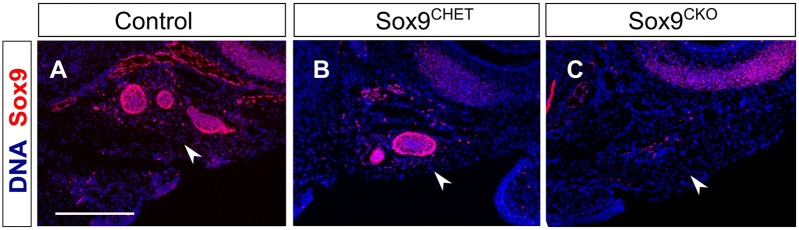
**Sox9 is required for parotid gland development.** (A-C) Sox9 immunofluorescence (red) in control (A), *Sox9^CHET^* (B) and *Sox9^CKO^* (C) parotid glands at E15.5. Arrowheads indicate the position of the parotid gland. DNA is shown in blue (DAPI). Scale bar: 250 μm (A-C).

Although the initial thickening was normal, the bud was smaller at E12.5 (Fig. S1B,D). This defect was more marked as the gland continued to develop with *Sox9^CKO^* SMGs failing to branch (Fig. 4A-C). Development of the submandibular and sublingual glands arrested at the bud stage at time points when control glands had undergone extensive branching (Fig. 4D,E). A delay in branching was also evident in the heterozygous *Sox9^CHET^* mice (*Sox9^flox/+^; K14-Cre^+^*) (Fig. 4B). The mesenchymal capsule that develops around the epithelial tissue still formed in the *Sox9^CKO^* mutants (Fig. 4D,E), however, as has been observed in *Fgf10* mutant mice (Wells et al., 2013). Similar to the SMG and SL, the PG was undetectable by E15.5 (Fig. 5) suggesting that Sox9 is required for the formation of all three major salivary glands.

As branching morphogenesis is a process that involves cleft formation and epithelial bud outgrowth through proliferation (Harunaga et al., 2011), we assessed cleft formation by morphological observation and laminin deposition (Fig. 4F; Fig. S2) and proliferation by detecting bromodeoxyuridine (BrdU) incorporation (Fig. 4G-I). Although some degree of variability was observed, approximately two-thirds of the *Sox9^CKO^* SMGs displayed no signs of cleft formation, i.e. no ingression of laminin into the bud (*P*<0.0001) (Fig. 4F; Fig. S2C) in contrast to controls, in which clefts were observed in every case (Fig. 4F; Fig. S2A). In the *Sox9^CKO^* SMGs in which a cleft formed, only a single cleft was observed, whereas in the wild type two or more clefts were evident (Fig. S2). In the lung, *Sox9* ablation has been reported to cause aberrant laminin deposition on the basal surface of the epithelium. However, in our mutant salivary glands close examination of laminin in epithelial cells revealed no obvious deposition on the basal surface compared with controls (Fig. S2A′-C′), suggesting key differences between the lung and salivary glands.

When proliferation was assessed, the ratio of cells that incorporated BrdU as a proportion of the total number of cells in the epithelium was reduced by approximately 25% in the *Sox9^CKO^* SMGs compared with the control glands at the bud stage (*P*<0.05) (Fig. 4G-I). The total number of epithelial cells was also reduced, by approximately 50%, in the *Sox9^CKO^* SMGs compared with that of controls at this stage (*P*<0.05) (Fig. 4L).

Having established that proliferation levels were significantly reduced in the mutant, we then investigated cell death in the glands. Apart from a few activated caspase 3^+^ cells at the site of ductal formation in both control and mutant glands (Fig. 4J,K, arrowheads) (Teshima et al., 2016b), no aberrant activation was detected in the distal compartment, suggesting that loss of *Sox9* does not lead to death of the epithelial cells of the gland.

**Fig. 6.**
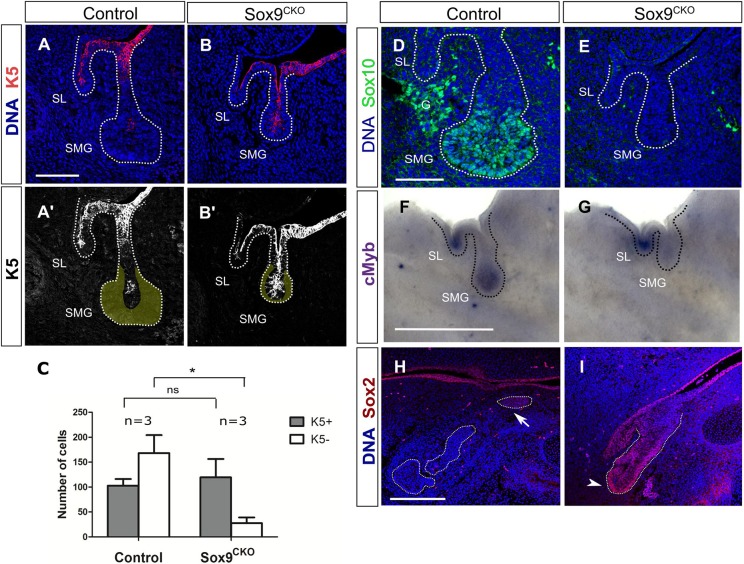
**Sox9 is required for the specification of distal epithelial progenitors.** (A-B′) Immunofluorescence for cytokeratin 5 (K5) (red) in control (A,A′) and *Sox9^CKO^* (B,B′) submandibular glands at the bud stage (E12.5). Yellow area in A′,B′ represents the K5^−^ distal epithelial cells. (C) Total number of K5^+^ and K5^−^ epithelial cells in control and *Sox9^CKO^* submandibular glands at the bud stage (E12.5). *P<0.01; ns, not significant. Error bars represent s.e.m. (D,E) Immunofluorescence for Sox10 (green) in control (D) and *Sox9^CKO^* (E) submandibular glands at the bud stage (E12.5). (F,G) *In situ* hybridisation for *Myb* in control (F) and *Sox9^CKO^* (G) submandibular glands at the bud stage (E12.5). (H,I) Immunofluorescence for Sox2 in control (H) and *Sox9^CKO^* (I) submandibular glands at the pseudoglandular stage (E13.5). Dotted lines (A-B',D-I) delineate the salivary gland epithelium. Arrow and arrowhead indicate the proximal and distal progenitors, respectively. DNA is shown in blue (DAPI) in A,B,D,E,H,I. G, ganglion; SL, sublingual gland; SMG, submandibular gland. Scale bars: 100 μm (A-B',H,I); 50 μm (D,E); 500 μm (F,G).

During branching morphogenesis (E13.5), epithelial progenitors express different markers depending on their location along the distal-proximal axis of the developing salivary gland. These distinct populations contribute to the formation of different epithelial structures (Matsumoto et al., 2016). Given that Sox9 is differentially expressed from E12.5 onwards, we assessed the expression of proximal (K5) and distal (Sox10, *Myb*) markers before the initiation of branching morphogenesis, and found that the epithelial cells could also be divided into two different populations at the bud stage, with proximal cells located at the stalk expressing K5 (Fig. 6A) and distal cells located at the tip of the endbud expressing Sox10 and *cMyb* (Fig. 6D,F). The early specification of the initial bud into distinct identities can be highlighted by dissecting the gland into distal and proximal compartments. The distal endbud goes on to branch in isolation, whereas the proximal stalk region fails to branch and has more limited growth (Fig. S3A-E). These data suggest that branching can initiate and progress independently of the proximal epithelium.

To understand the role of Sox9 in distal cell fate, we investigated the expression of Sox10 and *Myb* in *Sox9^CKO^* SMGs. Sox10 has been shown to be positively regulated by *Sox9* in the lacrimal glands (Chen et al., 2014), whereas Myb has been shown to inhibit acinar differentiation in SMGs (Matsumoto et al., 2016). Both Sox10 and *Myb* were at low or undetectable levels in the *Sox9^CKO^* SMGs (Fig. 6D-G) indicating loss of this progenitor population in the absence of *Sox9*. To examine the identity of the epithelial progenitors in the *Sox9^CKO^* SMGs, we investigated the expression of the proximal marker K5 (Fig. 6A,B). When the total number of cells was compared, the number of cells with a proximal identity remained the same, but the number of K5^−^ cells dramatically dropped (Fig. 6C). This suggests that, contrary to the distal progenitors, the proximal progenitors do not require Sox9 expression for their formation. To follow the proximal precursors at a later stage we then investigated the expression of another proximal marker, Sox2, at E13.5. Sox2 is normally expressed in the proximal epithelial progenitors in the ductal region of E13.5 control SMGs (Fig. 6H, arrow) (Lombaert et al., 2011). However, expression in the absence of *Sox9* was found throughout the epithelium including the tip of the truncated *Sox9^CKO^* endbud (Fig. 6I, arrowhead) suggesting that normal differentiation can proceed in the absence of *Sox9* in the remaining proximal progenitors. Altogether, these data illustrate the differential requirement of *Sox9* for the formation of the distal progenitors as opposed to the proximal progenitors.

### Conserved dependence of type II collagen on *Sox9* expression in salivary gland epithelium

**Fig. 7.**
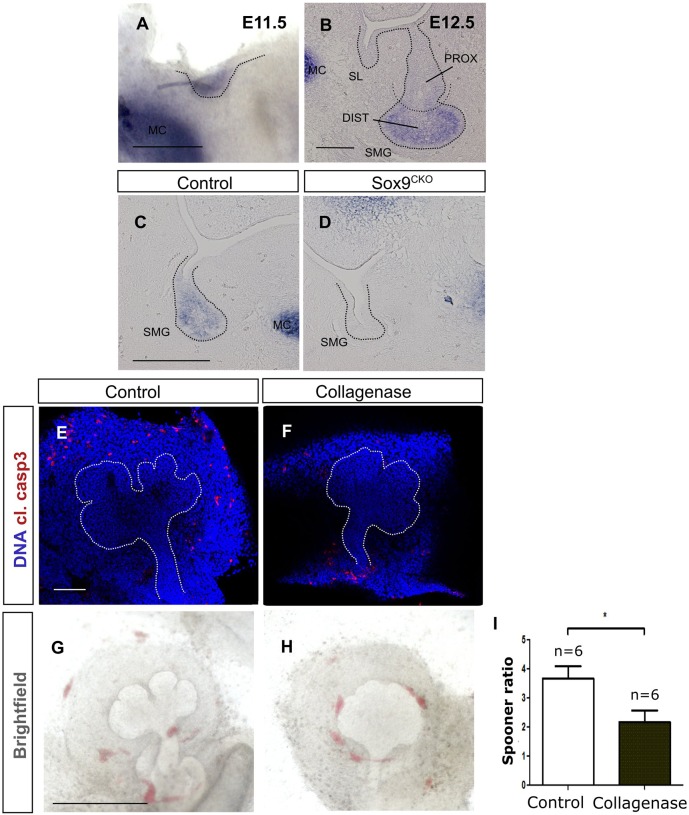
**Type II collagen (*Col2a1*) is expressed in the distal progenitors and acts downstream of Sox9 possibly by contributing to branching.** (A,B) *In situ* hybridisation for *Col2a1* at the placode (A) and bud stage (B). (C,D) *In situ* hybridisation for *Col2a1* at the bud stage (E12.5) in control (C) and *Sox9^CKO^* (D) submandibular glands. (E,F) Immunofluorescence for cleaved caspase 3 (red) in control (E) and collagenase-treated (F) submandibular gland explants. DNA is shown in blue (DAPI). (G,H) Brightfield images of control (G) and collagenase-treated (H) submandibular gland explants. (I) Spooner ratio of the number of buds produced in the control and collagenase-treated submandibular gland explants. *P<0.05. Dotted lines (A-H) delineate the salivary gland epithelium. Error bars represent s.e.m. DIST, distal; MC, Meckel's cartilage; PROX, proximal; SL, sublingual gland; SMG, submandibular gland. Scale bars: 250 μm (A); 50 μm (B); 100 μm (C-F); 500 μm (G,H).

In the mesenchyme, *Sox9* is part of a hierarchy of genes that control cartilage development (Bell et al., 1997). Some aspects of this pathway also appear to be conserved in epithelial tissues, for example with type II collagen being expressed in lung and lacrimal gland epithelium (Rockich et al., 2013; Chen et al., 2014). We therefore aimed to test whether type II collagen was also expressed in salivary gland epithelium. *Col2a1* was observed in the salivary gland epithelium from E11.5, overlapping with Sox9 expression (Fig. 7A, compare with Fig. 1B). As with Sox9, *Col2a1* was later restricted to the distal precursors (Fig. 7B). To test whether *Col2a1* expression was dependent on *Sox9*, we assessed expression in our conditional mutants (Fig. 7C,D). In the absence of *Sox9*, *Col2a1* expression was lost in the gland, suggesting a conserved relationship between these genes in both mesenchyme and epithelium (Fig. 7D).

To investigate whether the reduction of *Col2a1* expression could contribute to the branching defect observed in the *Sox9^CKO^* mice, submandibular glands were treated *ex vivo* with collagenase for 2 days (Fig. 7E-H). Collagenase treatment did not increase apoptosis in the epithelium, indicating no or low cytotoxic effects at this concentration (Fig. 7E,F). However, in agreement with previous observations from later stages (Nakanishi et al., 1986), collagenase treatment resulted in a reduction in branch formation (Fig. 7G-I). Thus, disruption of type II collagen in *Sox9^CKO^* salivary glands might contribute to the defect in epithelial branching morphogenesis.

### Fgf10 maintains Sox9 expression through the Erk pathway during SMG development

**Fig. 8.**
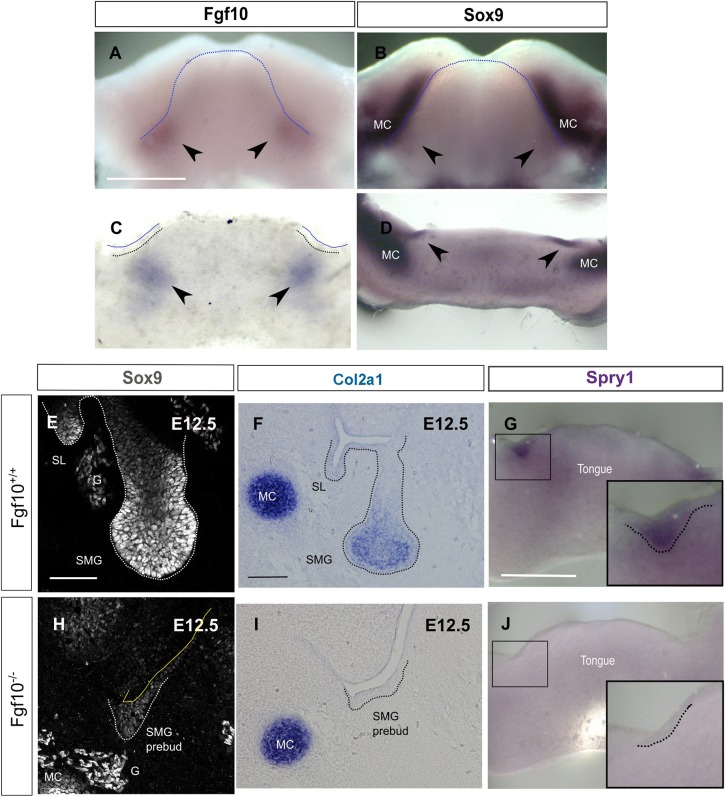
**Fgf10 maintains Sox9 expression during the initial stages of salivary gland development.** (A-D) *In situ* hybridisation for *Fgf10* (A,C) and *Sox9* (B,D) on E11.0 mandibles (A,B) and frontal mandibular slices (C,D). Arrowheads indicate the site of expression in submandibular glands. (E,H) Immunofluorescence for Sox9 in *Fgf10^+/+^* and *Fgf10^−/−^* submandibular glands at E12.5. (F-J) *In situ* hybridisation for *Col2a1* (F,I) and *Spry1* (G,J) in *Fgf10^+/+^* (F,G) and Fgf10^−/−^ (I,J) submandibular glands. Dotted lines delineate the tongue (A,B), the placode of the salivary glands (C,G,J) or the salivary gland epithelium (E,F,H,I). Boxes (G,J) indicate the placode of the developing submandibular glands, as magnified in insets. G, ganglion; MC, Meckel's cartilage; SL, sublingual gland; SMG, submandibular gland. Scale bars: 500 μm (A-D,G,J); 50 μm (E,H); 100 μm (F,I).

As Fgf signalling positively regulates Sox9 in other developing branching organs (Seymour et al., 2012; Chang et al., 2013; Chen et al., 2014), we hypothesised that Fgf10 might play a similar role in the SMGs. In keeping with this, qPCR analysis has previously shown upregulation of *Sox9* after addition of Fgf7 or Fgf10 to wild-type epithelial rudiments of SMG at the pseudoglandular stage (Lombaert and Hoffman, 2010). We first examined the expression of *Fgf10* and *Sox9* at the SMG initiation stage by *in situ* hybridisation (Fig. 8A-D). As previously described, *Fgf10* was expressed in the mesenchyme surrounding the site of placode formation (Fig. 8A,C) (Wells et al., 2013) whereas *Sox9*, as we have shown, was specifically expressed at the site of the epithelial thickening (Fig. 8B,D). Given the similar temporal and spatial localisation of *Fgf10* and *Sox9*, we were interested to see whether this pattern correlates with a positive regulation. Thus, we examined the expression of Sox9 in *Fgf10* null mice (Fig. 8E,H). *Fgf10* null mice fail to develop a bud and their development is arrested at the placode stage (Jaskoll et al., 2005). Sox9 was highly expressed in the bud of the *Fgf10*^+/+^ SMGs but it was severely reduced in the developmentally arrested placodes of the E12.5 *Fgf10* null SMGs. However, the mesenchymal expression of Sox9 in Meckel's cartilage and in the ganglion remained at the same levels (Fig. 8E,H), suggesting Sox9 in these tissues is not regulated by Fgf10. In addition, in keeping with the close relationship between *Sox9* and type II collagen, expression of *Col2a1* in the gland tissue was severely reduced at E12.5, with no effect on *Col2a1* expression in the adjacent Meckel's cartilage (Fig. 8F,I). Loss of Sox9 in the epithelium correlated with a reduction in the expression of *Spry1*, a readout of Erk signalling, suggesting that activation of Sox9 by Fgf10 acts through the Erk pathway during these initial stages of SMG development (Fig. 8G,J).

**Fig. 9.**
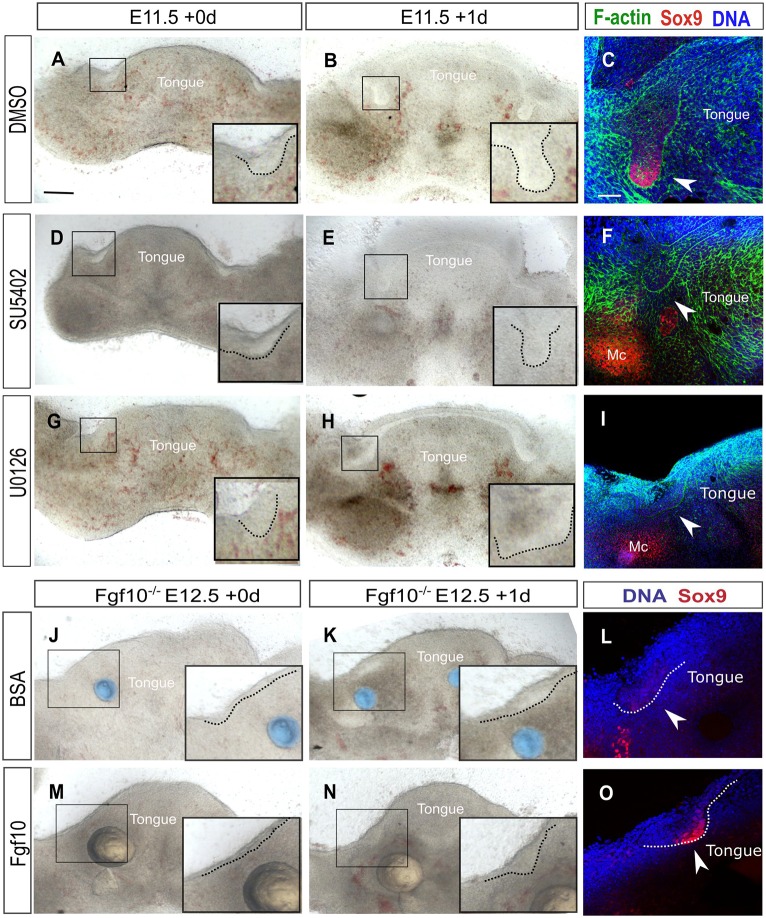
**Fgf** **receptor** **signalling maintains Sox9 expression through the Erk pathway.** (A,B,D,E,G,H) Brightfield images of wild-type mandibular slice cultures treated with DMSO (A,B), the Fgf receptor inhibitor SU5402 (D,E) or the Erk inhibitor U0126 (G,H). (C,F,I) Immunofluorescence for Sox9 (red) and F-actin (green) in DMSO- (C), SU5402- (F) and U0126- (I) treated mandibular slice cultures. (J,K,M,N) Brightfield images of *Fgf10*^−/−^ mandibular slice cultures treated with BSA-treated beads (blue) (J,K) or Fgf10-treated beads (pale yellow) (M,N). (L,O) Immunofluorescence for Sox9 in *Fgf10^−/−^* mandibles treated with BSA-treated beads (L) or Fgf10-treated beads (O). DNA is shown in blue (DAPI) in C,F,I,L,O. Boxes indicate the placode of the developing submandibular glands. Insets show higher magnifications of the boxed areas. Dotted lines outline the epithelium of the placodes. Arrowheads indicate the submandibular glands. Mc, Meckel's cartilage. Scale bars: 200 μm (C,F,I,L,O); 500 µm (A,B,D,E,G,H,J,K,M,N).

To study this positive regulation of Sox9 by Fgf10 further, we moved to an explant culture system. Mandibles were sliced frontally and slices with SMGs were cultured for 24 h (Fig. 9). In control cultures, the salivary gland tissue developed from a thickening to a bud and exhibited high levels of Sox9 (Fig. 9A-C). In contrast, slices cultured with SU5402, an inhibitor of the Fgf receptor signalling pathway, failed to develop a fully formed bud and Sox9 levels were undetectable (Fig. 9D-F), mimicking the *Fgf10* knockout phenotype. In contrast, Sox9 levels were maintained at high levels in the cultures in the absence of the inhibitor (Fig. 9C).

Fgf receptors signal through several transduction pathways the most common of which is the RAS-Erk pathway (Thisse and Thisse, 2005). To investigate which pathway controls Sox9 expression downstream of Fgf receptors, we inhibited the Erk pathway using the MAPK inhibitor U0126. Mandible slices were treated at E11.0 for 1 day with U0126 and DMSO-treated cultures were used as a control (Fig. 9G-I). Similar to the SU5402 treatment, the epithelium of the U0126-treated explants failed to form a fully developed bud (Fig. 9H) and to maintain Sox9 expression (Fig. 9I) suggesting that Fgf receptor signalling positively regulates Sox9 through the Erk pathway.

As Fgf10 is required to maintain Sox9, we went on to investigate whether exogenous Fgf10 treatment could restore Sox9 expression in *Fgf10* null SMG epithelium (Fig. 9J-O). As an Fgf10 source we used heparin-coated beads treated with Fgf10 to provide a localised supply of the protein (Fig. 9M,N); bovine serum albumin (BSA)-treated beads were used as a control (Fig. 9J,K). Beads were placed on E12.5 *Fgf10* null mandible slices in culture and the expression of Sox9 was assessed (Fig. 9L,O). The level of Sox9 expression was rescued in the Fgf10-treated slices compared with controls (Fig. 9L,O), further supporting the suggestion that Sox9 is positively regulated by Fgf10 in salivary glands.

### *Sox9* ablation does not lead to downregulation of *Etv5*

In the lacrimal glands, pancreas and kidney, Sox9 is involved in a positive-feedback loop with Fgf10 for further upregulation of Fgf signalling (Chen et al., 2014; Seymour et al., 2012; Reginensi et al., 2011), with *Etv5* expression, a downstream target of the Fgf receptor pathway, reduced in the *Sox9* mutant. To test whether Sox9 plays a similar role in salivary glands, we performed *in situ* hybridisation for *Etv5* (Fig. S4A,B) and also for *Fgf10* (Fig. S4C,D) on *Sox9* mutant glands. In contrast to the development of other branching organs, no detectable difference was found between the mutants and control for both *Etv5* (Fig. S4A,B) and *Fgf10* (Fig. S4C,D), indicating that Sox9 does not act in a positive-feedback loop with Fgf signalling in salivary glands. Salivary glands, therefore, appear to have distinct differences in Fgf signalling compared with other branching organs.

## DISCUSSION

Sox9 is a transcription factor involved in the development of many branching organs including pancreas, lacrimal glands, lungs and kidneys. Although salivary glands are also branching organs, the role of Sox9 during their development has not previously been addressed. Here, we have shown that Sox9 is expressed throughout the development of salivary glands from the salivary gland initiation stage to the fully differentiated adult salivary gland. These early Sox9^+^ epithelial cells are the progenitors of the entire salivary gland epithelium. In order to assess Sox9 function, we used the *K14* promoter to specifically ablate epithelial *Sox9* expression from the developing salivary glands. We demonstrated that *Sox9* is required for salivary gland morphogenesis by promoting the formation of the distal epithelial progenitor population, the presence of which is essential for subsequent branching. Abnormal branching and gland formation was observed in all three major *Sox9^CKO^* glands, the submandibular, sublingual and parotid. *Sox9* is therefore required for the development of all three major salivary glands, irrespective of whether the gland is mucous or serous.

### *Sox9* is required for the formation of distal epithelial progenitors and branching morphogenesis

Branching morphogenesis is a dynamic process that involves repetitive rounds of epithelial budding, clefting and epithelial outgrowth. This requires the coordination of different mechanisms, which includes ECM deposition, cell migration and epithelial proliferation (Harunaga et al., 2011). We have shown here that the mechanism of branch formation can be driven by the distal part of the epithelium alone (endbud) without the need of the proximal (stalk) epithelium. The branching defect observed in the *Sox9^CKO^* salivary glands is related to a failure in the specification of the distal epithelial population. Despite subtle differences in clefting, which could be attributed to differences in the number of Sox9^+^ cells that remained after recombination, all the *Sox9^CKO^* SMGs examined were arrested at the bud stage with an absence of the distal markers *Myb* and Sox10. Interestingly, this phenotype is specific to the salivary glands as *Sox9* ablation in other branching organs leads either to complete agenesis (lacrimal glands) (Chen et al., 2014) or to reduced branching (lungs, pancreas) (Chang et al., 2013; Rockich et al., 2013; Seymour et al., 2007), suggesting that the requirement for *Sox9* during development is specific to the branching organ. Despite the tissue-specific requirement for *Sox9*, we have shown that in salivary glands *Sox9* can regulate a similar subset of genes important for branching. This includes *Sox10* and *Col2a1*, which are also downregulated in the *Sox9^CKO^* lacrimal glands and lungs (Chen et al., 2014; Rockich et al., 2013).

Loss of type II collagen expression could contribute to the arrest in branch formation observed in the *Sox9^CKO^* SMGs as reduction of collagens with collagenase treatment in culture led to a loss of branching. In keeping with this, inhibition of collagenases has been shown to stimulate branching morphogenesis (Nakanishi et al., 1986). Our paper therefore provides a link between Sox9, distal progenitor formation and branching morphogenesis.

### Fgf10 signalling positively regulates *Sox9* expression through the Erk pathway

Sox9 has a distinct proximo-distal expression pattern from early bud stages; however, at the placode stage it is expressed throughout the epithelium. This change in expression might be driven by the changing pattern of *Fgf10* expression, which becomes more focused around the distal part of the gland as it develops. In the *Fgf10* null salivary gland, expression of Sox9 was lost at the late placode stage. In culture, Fgf7 has been shown to be able to strongly increase the expression levels of *Sox9* (Lombaert and Hoffman, 2010), but *in vivo* Fgf10 appears to be the dominant Fgf for Sox9 expression. The *Fgf10* null, however, had a more severe phenotype than the conditional *Sox9* mutant with an arrest at the placode stage. Although some of the phenotype in the *Fgf10* null might be generated by loss of Sox9, other genes are also likely to be affected. For example, inhibition of Fgf receptor signalling influences the activity of Wnt and Bmp signalling (Patel et al., 2011; Knosp et al., 2015; Hoffman et al., 2002).

*Fgf10* heterozygous mice are viable but have been shown to have smaller salivary glands (May et al., 2015). Interestingly, at E13.5 the *Sox9^CHET^* glands were smaller than the control littermates and had reduced numbers of branches, it would therefore be interesting to study whether the glands stay small or are rescued later in development.

In our culture experiments, we were able to rescue the expression of Sox9 in *Fgf10* null glands by addition of Fgf10 protein, implying that Sox9 is regulated by Fgf10 acting through the Erk pathway. Although loss of *Sox9* has been associated with a subsequent loss of Fgf signalling in many branching organs, we saw no such reduction in the salivary glands (Chen et al., 2014; Seymour et al., 2012; Reginensi et al., 2011). This implies that a positive-feedback loop between *Sox9* and *Fgf10* is not a universal part of branching morphogenesis. In the lungs, although *Etv5* is downregulated, *Fgf10* itself appeared to be upregulated (Chang et al., 2013). Again, we found no change in *Fgf10*, confirming that Sox9 does not appear to be able to influence Fgf signalling in salivary glands. Interestingly, although inhibition of Fgf10 and Erk signalling led to a loss of Sox9 in the gland epithelium, no change in Sox9 expression was observed in the neighbouring developing cartilage, showing that although some aspects of the cartilage pathway are preserved in the glands (Sox9 induction of type II collagen), the specific involvement of Erk signalling is unique to the glands.

**Fig. 10.**
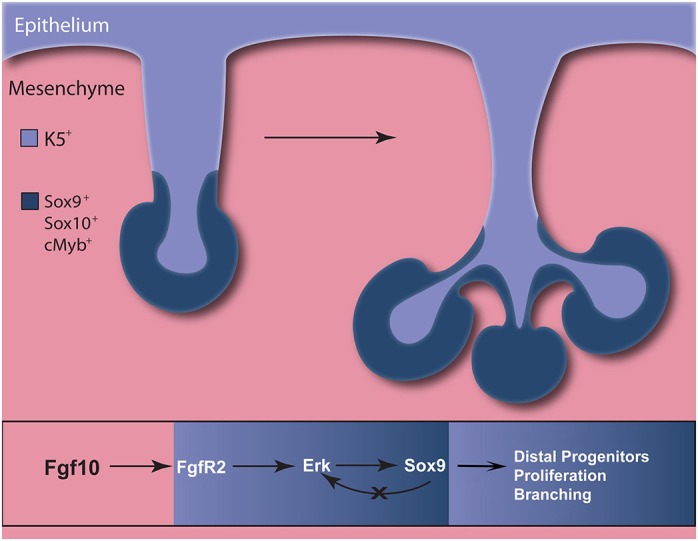
**Model of Fgf10 and Sox9 function during salivary gland budding and branching morphogenesis.** Sox9 is required for branching initiation by promoting the formation of distal epithelial progenitors and their proliferation. Mesenchymal Fgf10 maintains epithelial Sox9 expression during salivary gland development by activating the Erk pathway through Fgfr2.

The current results lead us to introduce a working model in which mesenchymal Fgf10 via the Fgf and Erk pathway, activates Sox9 expression in the epithelium. Sox9 promotes the formation and proliferation of distal epithelial progenitors, and in the absence of this population the gland is unable to undergo branching morphogenesis (Fig. 10). These results provide insights into the mechanisms of progenitor cell function underlying normal salivary gland morphogenesis and could prove useful in designing methods for regeneration of branching organs.

## MATERIALS AND METHODS

### Mouse strains and lineage tracing

*Sox9* floxed, *Fgf10* null and *K14-cre*, *Sox9-creERT2* and *Rosa-tdTomato* mice have been previously described (Kist et al., 2002; Min et al., 1998; Vasioukhin et al., 1999; Soeda et al., 2010; Madisen et al., 2010). For the lineage-tracing experiments, 75 mg tamoxifen/kg body weight was administered interperitoneally into E10.5 pregnant mice. The day of the vaginal plug was estimated as day 0.5 of embryonic development. All procedures and culling methods were compliant with UK Home Office regulations and with the approval of the King's College London Biological Safety committee.

### Histology, immunofluorescence and *in situ* hybridisation

Tissue was embedded in paraffin as previously described (May et al., 2015). Immunofluorescence was performed either on paraffin-embedded tissue or on whole-mount dissected embryonic salivary glands and explant cultures (Gaete et al., 2015). Primary antibodies and dilutions were used as follows: anti-Sox9 1:300 (AB5535, Millipore); anti-BrdU 1:500 (ab6326, Abcam), anti-Sox2 1:200 (#2748, Cell Signaling Technology); anti-Mist1 1:50 (sc-98771, Santa Cruz Biotechnology), for which signal was amplified with the TSA kit (PerkinElmer); anti-laminin 1:300 (L9393, Sigma); anti-K5 1:300 (119-13621, Cambridge Bioscience); anti-Sox10 1:100 (sc-365692, Santa Cruz Biotechnology) using the TSA kit; anti-cleaved caspase 3 1:200 (#9661, Cell Signaling Technology). *In situ* hybridisation was performed as previously described (Gaete et al., 2015). Plasmids for probe generation have been described previously: *Spry1* (Minowada et al., 1999), *Fgf10* (Bellusci et al., 1997), *Myb* (Matalová et al., 2011), *Etv5* (Hippenmeyer et al., 2002) and *Col2a1* (Ng et al., 1997).

### Proliferation and cell quantification analysis

For proliferation analysis, 20 mg BrdU per kg of pregnant mouse were injected intraperitoneally 30 min before harvesting. Tissue was then embedded in paraffin and processed for immunofluorescence. For BrdU immunofluorescence, samples were treated for 30 min with 2 M HCl at 40°C prior to the addition of primary antibody. The mean cell proliferation index (BrdU^+^/epithelial cells) for each gland was determined by analysing three different sections. For the cell quantification of epithelial progenitors, the section passing through the middle of the gland was quantified. Cells were quantified manually using the cell counter plug-in of Fiji/ImageJ (Schindelin et al., 2012). Results were plotted and statistically analysed using GraphPad Prism software. Data were analysed using a one-way ANOVA test apart from the cleft formation graph, which was analysed using the Chi-squared test. For all the quantification experiments, at least three independent biological replicates were used. Significance was taken as *P*<0.05 (*), *P*<0.01 (**) or *P*<0.001 (***).

### Explant culture

Mandibular slice cultures were performed as previously described (Wells et al., 2013; Li et al., 2016). For the bead experiment, two types of beads were used to help distinguish between the control and treated conditions. For the Fgf10-treated explants, heparin beads (Sigma, 100-200 mesh) were incubated overnight at 4°C with 100 μg/ml Fgf10 (R&D Systems). For the control, Affi-Gel blue beads (Bio-Rad,153-7302) were treated with 0.5% BSA. For inhibiting Fgf receptor signalling or the Erk pathway, explant cultures were treated with 2.5 μM SU5402 (Merck) or 5 μM U0126 (Cell Signaling Technology), respectively, made up in DMSO. Control cultures were treated with equivalent concentrations of DMSO (0.25% DMSO for the SU5402 and 0.5% DMSO for the U0126 experiment). For the collagenase treatment, whole E12.5 submandibular glands were dissected and treated for 2 days with 1 μg/ml collagenase, Type II (Thermo Fisher Scientific) and HBSS-treated glands were used as a control. Spooner ratios were calculated as the number of buds at the end of culture divided by the number of buds at the start of culture.
